# Extraction of phenol compound from *Mentha piperita* by ultrasonic waves based on a response surface methodology

**DOI:** 10.1002/fsn3.2467

**Published:** 2021-07-19

**Authors:** Sareh Roshanpour, Javad Tavakoli, Faranak Beigmohammadi, Shima Alaei, Amin Mousavi Khaneghah

**Affiliations:** ^1^ Faculty of Agriculture Department of Food Science and Technology Kermanshah Branch Islamic Azad University Kermanshah Iran; ^2^ Faculty of Agriculture Department of Food Science and Technology Jahrom University Jahrom Iran; ^3^ Plant Biotechnology Research Center Kermanshah Branch Islamic Azad University Kermanshah Iran; ^4^ Faculty of Food Engineering Department of Food Science and Nutrition University of Campinas (UNICAMP) Campinas Brazil

**Keywords:** antioxidant activity, extract, phenolic compound, optimization

## Abstract

In this study, optimization of the extraction of phenol compounds from *Mentha piperita* using ultrasonic waves with response surface methodology (RSM) was assessed. In this regard, a central composite design with three independent variables of time (5, 27.5, and 50 min), temperature (25, 45, and 65°C), and concentrations of ethanol in the water–ethanol solution (0%, 50%, and 100%) was used. Besides, the antioxidant activity tests (DPPH radical scavenging assay, ferric reducing antioxidant power [FRAP], and oxidative stability indexes [OSI]) were examined. Significant effects of independent variables on the extraction of phenol compound, DPPH radical scavenging power, and OSI of *M. piperita* extract, with the regression coefficients of 0.89, 0.92, and 0.94, respectively, were noted. However, no significant difference in terms of the FRAP among different treatments was noted. Also, the best antioxidant activity of *M*. *piperita* was obtained by using the ultrasonic wave for 50 min at 65°C and 59.6% v/v ethanol/water solution. While the findings of RSM confirmed the experimental results, due to the favored properties of *M*. *piperita* extract by the proposed method, further research to investigate possible applications in the food industry is recommended.

## Graphical abstract

In this study, optimization of the extraction of phenol compounds from Mentha piperita by using the ultrasonic waves with the aid of response surface methodology (RSM) was assessed. In this regard, a central composite design with three independent variables of time (5, 27.5, and 50 min), temperature (25, 45, and 65°C), and concentrations of ethanol in the water–ethanol solution (0%, 50%, and 100%) was used.

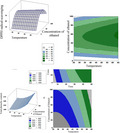

## 1 INTRODUCTION

The use of natural antioxidants is increasing because of their beneficial effects on human health (Akbarirad et al., 2016). However, the main associated shortcoming with using these antioxidants is the high cost of their extraction. In this regard, various investigations have been carried out regarding the extraction of these compounds with the aid of traditional methods (Soxhlet, mechanical, and Solvent extraction) and modern methods (microwave and ultrasonic) (Roshanpour et al., 2021; Farahmandfar et al., 2019; Farahmandfar et al., 2019; Hashemi et al., 2018; Hashemi et al., 2017; Tavakolpour et al., 2017; Akbarirad et al., 2016; Hashemi et al., 2013), while the employing of conventional methods due to long processing time, high consumption of solvent, and decomposition antioxidant compound due to uncontrollable temperature will be limited (Martins et al., 2013; Rajaee et al., 2010). In this regard, the optimization of antioxidant compounds extraction by newly introduced techniques attracted considerable attention. Among them, the application of ultrasound technology for natural antioxidant extraction has great potential in the herbal and food industries (Deng et al., 2017; Zhang et al., 2019), while in the food industry, low‐frequency waves (18–100 kHz) are the most commonly used waves for extraction of natural antioxidants from different sources (Arteaga‐Crespo et al., 2020; Farahmandfar, Asnaashari et al., 2019; Farahmandfar, Esmaeilzadeh Kenari, et al., 2019; Pandeya et al., 2018; Roshanpour et al., 2021; Zhang et al., 2019). In a study, Tavakoli et al., (2017) investigated the effect of the ultrasonic process on the antioxidant activity of the extract obtained from the *Cucurbita Pepo* peel extract. Their findings demonstrated that ethanol/water extract (5:1 v/v) combined with the ultrasonic process has a higher antioxidant activity due to the higher extraction performance for antioxidants. According to Hashemi et al. (2016), the application of ultrasonic waves enhances the efficiency and quality of the extracted oil of *Pistacia Khinjuk* hull, which can be archived in a lower extraction time while compared with the conventional methods.

The most commonly used method for optimizing phenols extraction and antioxidant activity is response surface methodology (RSM) (Miamoto et al., 2020; Martins et al., 2013; Bezerra et al., 2008). In this method, the effect of the single independent variable or some independent variables together on the results is analyzed (Lopez et al., 2011). In a study, the best antioxidant activity of the wheat germ and its bran was achieved at an ethanol concentration of 53%, 61°C, and 64 min and ethanol concentration of 49%, 64°C, and 60 min, respectively (Liyana‐Pathirana & Shahidi, 2005).

*Lamiaceae* family is one of Iran's most critical growing plants, which are rich in essential oil and terpenoid (Roshanpour et al., 2021). *Mentha* genus, mainly *M*. *spicata*, *M*. *piperita*, and *M*. *longifolia*, are some of the most critical aromatic herbals belonging to the *Lamiaceae* family, a domestic plant of Iran (Dzamic et al., 2010). Due to the good antioxidant activity of their methanolic and aqueous extracts from leaf, pedicle, and flower, rich in phenolic compounds, they are widely used for pharmaceutical, food, and cosmetics purposes (Abootalebian et al., 2016; Benabdallah et al., 2016; Dorman et al., 2003; Bazrafshani et al., 2019; Yadegarinia et al., 2006; Rajkumar et al., 2019; Yu et al., 2018). In this context, the relatively high antioxidant activity of essential oil and extract of *M*. *piperita*, while compared with synthetic antioxidants such as BHT, was demonstrated by (Singh et al., 2011).

Therefore, in the current study, the extraction process and chemical properties, mainly antioxidant activity of *M*. *piperita* by sonication using different ethanol–water solvent ratios, temperatures, and times, were investigated by employing a response surface methodology.

## 2 MATERIALS AND METHOD

### 2.1 Materials

The *M*. *piperita* was obtained from Shiraz Agricultural Research Center. After manually cleaning, it was dried in the shadow (20°C), and afterward, it was milled (Mullinex Depose‐Brevete S.G.C.G) to get the fine powder. The prepared powders were kept at 4°C until the day of the experiments.

The soybean oil without antioxidant added was supplied by Narges Shiraz Company. All standards and solvents chemicals, such as ethanol (95%) and n‐hexane (anhydrous, 95%), in the analytical grade were purchased from Merck.

### 2.2 Preparation of *M. piperita* extract

To optimize the condition, 50‐gram powder of *M*. *piperita* was dispersed in 250 ml of ethanol/water (0:100, 50:50, and 100:0) solvent. Then, the Erlenmeyer flask of the samples was placed in the ultrasonic bath (DT 102H, Bandelin). To find the optimum extraction condition with ultrasonic waves, different variables consisting of a ratio of ethanol to water (0:100, 50:50, and 100:0), the temperature (25, 45, and 65°C), and time (5, 27.5, and 50 min) were investigated. Then, the prepared solution was filtered via Whatman filter paper. The filtered solution exposed vacuum conditions until the complete evaporation of solvents. The prepared samples were kept at −18°C at the freezer until the experiments (Farahmandfar, Asnaashari, et al., 2019; Hammi et al., 2015).

### 2.3 Total polyphenol compounds assessment

The amount of polyphenol compounds were measured by Folin & Ciocalteu′s phenol reagent. The amount of polyphenol compounds is reported in milligram gallic acid on 100 g of extracted samples, based on the standard curve of gallic acid (Sfahlan et al., 2009; Tavakoli et al., 2019).

### 2.4 DPPH radical scavenging assay

The method of Yim et al. (2012) [19] was used for the DPPH radical scavenging capacity of extracted samples. After putting the samples in dark condition for 30 min at ambient temperature, the spectrophotometric assay of the samples was done for the wavelength of 517 nm. The antioxidant activity of the samples was calculated via the following equation (Tavakoli, Hajpour Soq, et al., 2019; Tavakoli, Sedaghat, et al., 2019; Yim et al., 2012):

(1) $$\%A=\left[\left[1‐\frac{A_{s}}{A_{c}}\right]\right]\times 100$$

A is the percentage of scavenging of DPPH radicals, As is the absorbance of the samples, and Ac is the absorbance of the control sample.

### 2.5 The ferric reducing antioxidant power

The ferric reducing‐antioxidant power (FRAP) test was conducted. Acetate buffer (0.3 M, pH 3.6) was prepared by dissolving 3.1 g C_2_H_3_O_2_Na. 3H_2_O and 16 ml of acetic acid in 1 L of distilled water. TPTZ (2,4,6‐tripyridyl‐S‐triazine) solution was prepared by dissolving 23.4 mg of TPTZ in 7.5 ml of 40 mM HCl solution. Ferric solution (20 mM) was prepared using FeCl_3_ 6H_2_O. The final working FRAP reagent was prepared freshly by mixing acetate buffer, TPTZ, and ferric solutions at a ratio of 10:1:1. In brief, a 900 ml FRAP working reagent was mixed with 90 ml distilled water and was warmed to 37°C in a water bath. The reagent blank reading was recorded at 595 nm, followed by adding 30 ml of sample solutions (100 mg in 10 ml of n‐hexane). The absorbance was taken at 595 nm, against the blank solution. A standard curve was prepared using different concentrations of FeSO_4_. 7H2O (200–2000 mmol/L). All solutions were freshly prepared. The results were expressed in mmol Fe_2_/L (Razali et al., 2012; Tavakoli, Hajpour Soq, et al., 2019; Tavakoli, Sedaghat, et al., 2019).

### 2.6 Oxidative stability index measurement

A Metrohm Rancimat model 743 was used to determine the antioxidative power of the extract samples in soybean oil. In this regard, 3 g antioxidant‐free soybean oil with 1,500 ppm of the extracted samples at 110°C with airflow speed of 15 L per hour was employed (Tavakoli et al., 2016). The antioxidative power of extracts was compared based on the OSI of different oil samples.

### 2.7 Statistical design and optimization of the process

Experimental design, analysis of results, and optimization of the extraction conditions were done via RSM using Minitab software. In this study, a central composite rotatable design with three independent variables, four dependent variables, three levels (The experimental design has three invoices level coding of −1, 0, and 1, respectively belong to low, middle, and high‐level coding), and 3 replications was approached. The independent variables consist of exposure time (X_1,_; 5, 27.5, and 50°C), temperature (X_2_; 25, 45, and 65 min), and the ratio of ethanol to water (X_3_; 0%, 50%, and 100%), and dependent variables consist of total polyphenol compounds, DPPH radical scavenging, FRAP, and OSI measurement. The domain of independent variables was selected based on the preliminary experiments. The results of central composite rotatable design were fit to the below quadratic polynomial:

(2) $$Y=\beta_{0}+\sum_{i}^{3}=1\beta_{i}X_{i}+\sum_{i}^{3}=1\beta_{\mathrm{ii}}X_{i}^{2}+\sum_{i}^{n}=0\sum_{j}^{3}=2\beta_{\mathrm{ij}}x_{i}x_{j}$$

In this model, β_0_, β_i_, β_ii_, and β_ij_ are the regression coefficients for the intercept, linear, quadratic, and interaction terms, respectively.

## 3 RESULTS AND DISCUSSION

### 3.1 Assessment of extraction model by RSM

Assessment of the extracted RSM results and comparison with different regression models showed that for all of the experimental data of this study, a significant difference between the quadratic polynomial model and other models was noticeable. The suitability of the models was probed via the study of *F* test, lack of fit, regression coefficient, predicted regression coefficient, adjusted regression coefficient, and *p*‐value. Among the various independent variables, the most significant sum of squares is selected as the most influential parameter (Myers et al., 2016). As shown in the variance analysis results (Table 1), the proposed model for extraction has the lowest *p*‐value level while the regression coefficient was higher than 0.8. Generally, the results of the analysis of variance demonstrated a good correlation between responses and independent parameters (temperature, time, and ethanol concentration) in the extraction of phenolic compounds and OSI of *M*. *piperita* extracts with corresponding values of 0.89 and 0.94 for regression coefficients, respectively. Also, a good correlation was noted between the independent parameters of temperature and ethanol concentration and DPPH radical scavenging capacity, with a regression coefficient of 0.92, while the time parameter was nonsignificant in this test.

**TABLE 1:** Analysis of variance of the quadratic model adjusted for total phenolic compounds, oxidative stability index (OSI), DPPH radical scavenging assay, and FRAP test

Source	Total phenolic compounds					Source	Oxidative stability index (OSI)				
	DF	Sum of Square	Mean Square	F‐Value	*p*‐value		DF	Sum of Square	Mean Square	F‐Value	*p*‐value
Model	6	161,391	26,898.5	67.15	0	Model	7	31.5698	4.51	111.48	0
Linear	3	125,328	41,775.8	104.28	0	Linear	3	5.034	1.678	41.48	0
X1	1	56,180	56,180	140.24	0	X1	1	0.4488	0.4488	11.09	.002
X2	1	60,756	60,755.7	151.66	−0	X2	1	1.4362	1.4362	35.5	0
X3	1	10,078	10,077.6	25.16	0	X3	1	3.4993	3.4993	86.5	0
Square	2	7,675	3,837.6	9.58	0	Square	2	22.9019	11.451	283.05	0
X12	1	3,709	3,709.4	9.26	.004	X22	1	0.2163	0.2163	5.35	.025
X22	1	5,467	5,467.1	13.65	.001	X32	1	17.4566	17.4566	431.51	0
2‐Way Interaction	1	9,003	9,003.5	22.47	0	2‐Way Interaction	2	0.442	0.221	5.46	.007
X1 X2	1	9,003	9,003.5	22.47	0	X1 X2	1	0.229	0.229	5.66	.021
Error	53	21,232	400.6			X2 X3	1	0.2131	0.2131	5.27	.026
Lack of fit	12	19,087	1,590.6	30.41	0	Error	52	2.1037	0.0405		
Pure Error	41	2,145	52.3			Lack of fit	11	1.6102	0.1464	12.16	0
Total	59	182,623				Pure Error	41	0.4935	0.012		
R2	0.8837					Total	59	33.6734			
Adj. R2	0.8706					R2	0.9375				
Pred. R2	0.8485					Adj. R2	0.9291				
						Pred. R2	0.9196				

Source	DPPH radical scavenging assay					Source	FRAP test				
	DF	Sum of Square	Mean Square	F‐Value	*p*‐value		DF	Sum of Square	Mean Square	F‐Value	*p*‐value
Model	4	5,998.8	1,499.7	151.82	0	Model	4	5,998.8	1,499.7	151.82	0
Linear	2	949.12	474.56	48.04	0	Linear	2	949.12	474.56	48.04	0
X1	1	446.31	446.31	45.18	0	X1	1	446.31	446.31	45.18	0
X3	1	645.9	645.9	65.39	0	X3	1	645.9	645.9	65.39	0
Square	1	4,759.2	4,759.2	481.79	0	Square	1	4,759.2	4,759.2	481.79	0
X32	1	4,759.2	4,759.2	481.79	0	X32	1	4,759.2	4,759.2	481.79	0
2‐Way Interaction	1	78.86	78.86	7.98	.007	2‐Way Interaction	1	78.86	78.86	7.98	.007
X2 X3	1	78.86	78.86	7.98	.007	X2 X3	1	78.86	78.86	7.98	.007
Error	55	543.29	9.88			Error	55	543.29	9.88		
Lack of fit	14	458.57	32.75	15.85	0	Lack of fit	14	458.57	32.75	15.85	0
Pure Error	41	84.73	2.07			Pure Error	41	84.73	2.07		
total	59	6,542.1				total	59	6,542.1			
R2	0.9170					R2	0.9170				
Adj. R2	0.9109					Adj. R2	0.9109				
Pred. R2	0.9036					Pred. R2	0.9036				

The effect of the independent parameters on the FRAP test was nonsignificant (*p*‐value >.05). Therefore, for this test, an analysis of RSM was not investigated (Table 1).

### 3.2 Analysis of RSM related to the effect of different factors on the polyphenolic compounds extraction and antioxidant activity

#### 3.2.1 Determination of optimal extraction conditions of phenolic compounds

Polyphenols are one of the most commonly investigated antioxidant compounds in plants, and their health effects have been confirmed. Based on the variance analysis, a quadratic polynomial fitting with a regression coefficient of 0.89 was associated with the extraction of phenol compound and independent parameters. Equation 3 is an appropriate model proposed by RSM:

(3) $$\text{TP}=1620.4+1.814\;X_{1}‐3.98\;X_{2}+0.3434\;X_{3}‐0.0336\;X_{1}^{2}+0.0540\;X_{2}^{2}+0.03907\;X_{1}*X_{2}$$

Where X_1_, X_2_, and X_3_ are time, temperature, and ethanol concentration, respectively.

**FIGURE 1:**
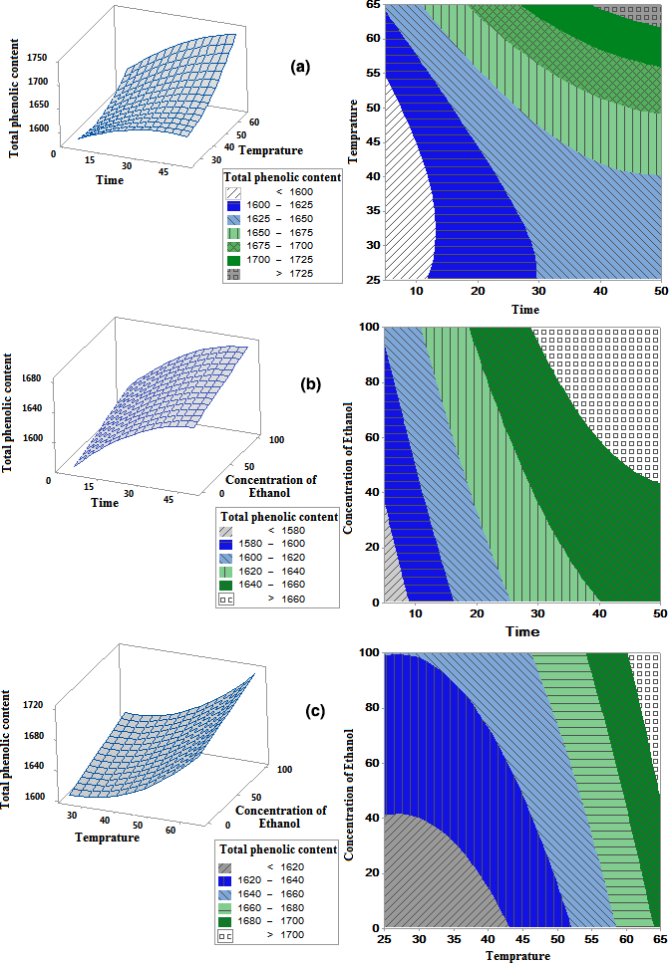
Response and contour plot for the effect of different parameters (time [min] and temperature [°C] (a), time [min] and concentration of ethanol [%] (b) and temperature [°C] and concentration of ethanol [%] (c)) on the amount of total phenolic content (mg/100 g)

This model shows the temperature and time had a significant quadratic, linear effect on the response of phenol extraction. Also, it was observed that ethanol concentration only had a linear effect on the response of polyphenolic compounds extraction. Therefore, the effect of time and temperature parameters on the extraction of the phenol compound of *M*. *piperita* was higher than the corresponding value for ethanol concentration. Figure 1 indicated the response surface and contour curves of the interaction effects of the independent variable (In binary form) on the efficiency of phenol extraction from *M*. *piperita*.

Probing the effect of two variables of time and temperature shows the optimum exposure time (with a significant impact on the extraction time) to the ultrasonic vibration was 50 min while the optimum temperature was 47.6°C (Figure 1A). Elaborating the time causes more interactions between soluble and solvent, which offers the more effective constituents of the plant to penetrate the solvent (Hossain et al., 2012). However, on an industrial scale, reducing the extraction time is necessary for economic profit. Pinelo et al. (2005) stated that increasing the extraction time affected our results by extracting polyphenolic compounds from berry and grape pomace. In other studies, the highest extraction of phenolic compounds was achieved after 45–90 min by ultrasonication, and continuing the process at higher times resulted in lower extraction efficiency and further degradations of these compounds (Huang et al., 2009; Wang et al., 2008). Temperature also significantly affects the extraction process by increasing the activation energy (Farizadeh et al., 2009). According to Oancea et al. (2012), the increasing temperature may decrease the extraction performance of phenolic compounds (Oancea et al., 2012). Also, Ya‐Qin and Jian‐Chu's (2009) results showed that an increase in the extraction of phenol compounds from citrus peel was achieved; however, in higher temperatures, the reversal trend was observed due to thermal decomposition or polymerization of polyphenol compound with themselves.

The extraction rate of phenolic compounds depends on temperature, time, and solubility (Yang et al., 2013). In this regard, some other factors affect the process of ultrasound extraction, including solvent polarity and volume of solvent. Figure 1B shows the simultaneous effect of time and ethanol concentration on the efficiency of phenol extraction from *M*. *piperita*, demonstrating that the optimum extraction condition was 50 min of exposure to the ultrasonic with an ethanol concentration of 55.1%. Similar to temperature, with an increase in the ethanol concentration in the ethanol/water solution, the efficiency of extraction from *M*. *piperita* was increased. However, with further increases in ethanol concentration, a decreasing trend was observed. Rahimi‐Panah et al. (2010) studied the optimum condition for phenol compound extraction from walnuts green peel and stated that an increase in the extraction time caused an increase in the concentration of extracted phenol compound while increasing in the percentage of ethanol showed a reverse effect. In this context, based on their findings, by decreasing methanol concentration from 100% to 60%, the amount of extracted phenolic compounds was increased. In another investigation, the optimum condition for phenol extraction from the whole wheat and wheat bran was correlated with two extraction conditions of 64 and 60 min and ethanol concentration of 53% and 49%, respectively (Liyana‐Pathirana & Shahidi, 2005). Increasing the extraction time and relative concentration of ethanol and water in the solvent resulted in the highest extraction of phenolic compounds. Figure 1C shows the two‐way interaction of temperature and ethanol concentration on the effectiveness of phenol compound extraction. According to Silva et al. (2007), which studied the optimization of the phenol compound extraction from the leaves of Inga edulis via RSM, the extraction of the phenolic compounds increased as a result of the increments in the temperature and ethanol concentration.

##### Finding the optimum phenol compound extraction condition via consideration of time, temperature, and ethanol concentration variable

**FIGURE 2:**
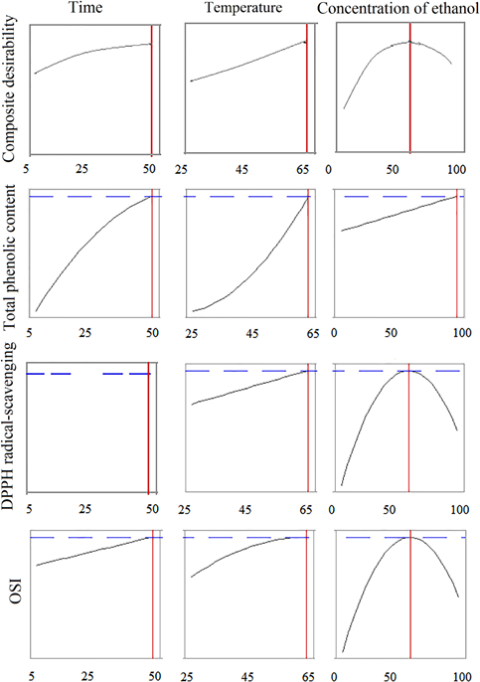
Optimum conditions of phenolic compounds extraction, DPPH radical scavenging and oxidative stability index, and composite desirability of mention assay under three independent parameters (time [min], temperature [°C], and concentration of ethanol [%])

Response surface methodology analysis of the data for optimization of three independent variables for phenol compound extraction indicates that a combination of 50 min, 65°C, and ethanol concentration of 100% is the optimum extraction condition (Figure 2). While the phenolic compounds are not extractable from the *M*. *piperita* tissue at low temperatures and early extraction time, increasing temperature with further changes (deformation) in the plant tissue enhances the phenol extraction. Also, the temperature increment causes the breakdown of the complex polyphenol compound to more minor phenol compounds. While due to their smaller size, they can extract more accessible from the plant tissue than complex polyphenol. In contrast, in the extraction process of phenolic compounds with a simple structure, the increasing temperature can cause some decompositions in the structure of these compounds.

Evaluation of the effect of ethanol concentration demonstrated due to the polar composition of *M*. *piperita* phenol compounds. They dissolve better in ethanol in comparison with water. Amyrgialaki et al. (2014) indicated that the use mixture of ethanol and water is one of the most suitable practices for extracting phenolic compounds. Water acts as a plant tissue swelling agent, while ethanol is a fragile graft of soluble material and plant matrix (Şahin & Şaml, 2013).

In this context, Tabaraki and Nateghi (2011) studied the optimum condition of phenol extraction from rice bran via ultrasonic treatment. Their findings show the optimum extraction condition was achieved at 51–54°C for 40–45 min combined with 65%–67% ethanol concentration. In another study about extraction phenol compound from the green peel of pistachio via ultrasonic vibration, the results showed the optimum condition was 20% of ethanol, 65°C, and 25 min exposure time (Rajaee et al., 2010). Also, an investigation regarding the extraction of phenol compounds from pennyroyal explored that by increasing the temperature, extraction time, and reduction of pH, higher performance for the extraction was achieved. In contrast, the optimum extraction condition was noted as 35–55 min, 35–45°C, and the pH of 6.0–6.5 (Heydari Majd et al., 2014). Upadhyay et al. (2015) investigated the effect of the ultrasonic process on the quality of *Ocimum tenuiflorum* leaf extract by response surface methodology and found out that the best treatments consisted of ethanol concentration of 55.34% and 11.71 min. Also, Arteaga‐Crespo et al. (2020) reported that the optimal levels of extraction of phenolic antioxidants from *Ilex guayusa Loes*. Leaves using response surface methodology (3.38 g gallic acid equivalents/100 g d.w.) were ethanol/water ratio of 76.8/23.2 v/v, 60°C, and 29.9 min.

#### 3.2.2 Optimization of DPPH radical scavenging

DPPH radical scavenging assay is one of the reliable assays used to investigate the antioxidant activity property of the extracted solution (Farahmandfar, Asnaashari, et al., 2019; Tavakoli, Hajpour Soq, et al., 2019). The variance analysis of the DPPH radical scavenging of independent variables indicated the regression coefficient of 0.92 for fitting with the quadratic equation from RSM:

(4) $$\text{DPPH = 45}.59+0\text{.0825}\;X_{2}+0.7437\;X_{3}‐0.007322\;X_{3}^{2}+0.001649\;X_{2}*X_{3}$$

This model shows temperature and ethanol concentration variables, significantly affecting DPPH radical scavenging, while all independent variables affect phenol compound extraction. It was also found that the two parameters abovementioned had both linear and interaction effects on the response of DPPH. However, the quadratic effect was only related to the concentration of ethanol. Therefore, the ethanol concentration had a greater effect on the DPPH radical scavenging of *M*. *piperita* extract while compared with the time.

**FIGURE 3:**
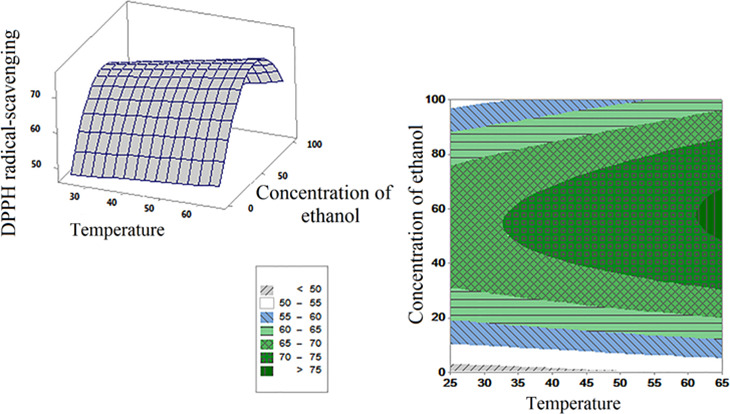
Response surface and contour plot for the effect of different parameters (temperature [°C] and concentration of ethanol [%]) on the amount of DPPH radical scavenging power (%)

Figure 3 shows the response surface and contour curves of two temperature and ethanol concentration variables on the DPPH radical scavenging of *M*. *piperita* extract. As can be seen, 65°C and ethanol concentration of 58.6% was the optimum condition for DPPH radical scavenging. Therefore, the best results were obtained at ethanol concentrations above the central point and at the highest temperature (unlike the extraction of phenolic compounds that observed optimum point at the highest temperature, time, and ethanol concentrations).

Antioxidants incorporating a higher concentration above the optimum concentration can cause peroxidase activity instead of antioxidant activity (Tavakoli et al., 2017). These findings are valuable to investigate the best antioxidant activity by consuming less ethanol which reduces extraction costs (Koocheki et al., 2012). Due to variety in extraction parameters (temperature, time, type of solvent, and solvent concentration), the structure of phenolic compounds extracted can be altered. Therefore, their antioxidant activity may be altered (Ilaiyaraja et al., 2015; Wei et al., 2015).

Morelli and Prado (2012) studied the optimum condition for DPPH radical scavenging of red grape extract via ultrasonic and reported that the 50°C and concentration of 60% ethanol and 20 min was the best condition. In another study, regarding the DPPH radical scavenging of olive leaves extract via ultrasonic, the optimum results were summarized as 60 min, 50% ethanol concentration, and solvent ratio to dry material 10:0.5 (ml/gr) (Şahin & Şaml, 2013). Lai et al. (2013) studied the optimum extraction condition of antioxidants from Black Soybean (*Glycine max var*), while 30°C, 32.13 min, and solvent ratio to dry material 29.19:1 was the optimum condition. Miamoto et al. (2020) evaluated the optimization of the extraction of polyphenols and antioxidant capacities from two types of *Solanum gilo Raddi* using response surface methodology. The results showed that the most important variable in the extraction procedure was the ethanolic degree, while the best ethanol degree was 61% for TPC and 65% for DPPH radical scavenging. According to findings of another investigation, the highest DPPH radical scavenging of the extract obtained from stem, leaves, and aerial parts with the aid of an ultrasonic process using response surface methodology was 10.66, 47.22, and 34.74%, respectively, which was observed at 93°C and 10 min (Irakli et al., 2018).

#### 3.2.3 Optimization of OSI (Rancimat test)

Rancimat test is usually using for investigating oxidative stability of the base of the oil on the changes in electrical conductivity due to the production of volatile acids (Farahmandfar, Esmaeilzadeh Kenari, et al., 2019; Tavakoli et al., 2016). Therefore, it was used to find the optimum extraction condition for reaching the highest antioxidant activity. For this reason, the extract was added to the antioxidant‐free refined soybean oil with a concentration of 1,500 ppm to investigate the related effect on the OSI of soybean oil at a temperature of 80°C. The adding of the *M*. *piperita* extracts, which were prepared via different conditions into the soybean oil, increased the OSI (Table 2).

**TABLE 2:** Comparison of the observed value and predicted data by central composite rotatable design equations of response surface methodology

Runs	Independent variable			Dependent variable						FRAP test
	Time (min)	Temperature (ºC)	Concentration of ethanol (%)	Total phenolic content (mg/100gr)		DPPH radical scavenging (%)		Oxidative stability index (hr)		
	X_1_	X_2_	X_3_	Observed value	Predicted value	Observed value	Predicted value	Observed value	Predicted value	Observed value
1	50	65	50	1,750 ± 5	1,740.6	78.32 ± 0.32	75.19	5.96 ± 0.11	5.67	68.38 ± 0.42
2	0	45	5	1,545 ± 5	1,567.6	50.14 ± 1.14	49.30	3.77 ± 0.05	3.76	44.18 ± 0.28
3	50	25	50	1,625 ± 5	1,627.3	61.31 ± 0.25	68.60	4.81 ± 0.06	5.11	54.39 ± 0.39
4	0	25	50	1,600 ± 8	1,610.1	50.22 ± 0.3	47.65	3.80 ± 0.10	3.65	47.07 ± 0.93
5	50	25	27.5	1,588 ± 8	1,623.1	64.95 ± 0.85	68.59	4.9 ± 0.06	5.09	55.31 ± 1
6	100	65	50	1,730 ± 5	1,757.8	60.6 ± 0.4	62.83	4.61 ± 0.10	4.84	52.88 ± 0.53
7	0	65	50	1,720 ± 10	1,723.4	50.84 ± 0.84	50.95	3.90 ± 0.04	4.03	46.28 ± 0.28
8	50	45	50	1,680 ± 8	1,662.3	75 ± 0.38	71.90	5.66 ± 0.02	5.54	67.82 ± 0.82
9	50	45	27.5	1,650 ± 8	1,640.6	77.42 ± 0.32	71.89	5.59 ± 0.03	5.42	64.37 ± 0.73
10	100	45	27.5	1,639.7 ± 13	1,657.8	55.39 ± 0.39	57.88	4.48 ± 0.02	4.51	54.57 ± 0.55
11	0	25	5	1,595 ± 5	1,567.7	48.59 ± 0.56	47.65	3.68 ± 0.03	3.60	48.52 ± 0.20
12	100	25	27.5	1,649 ± 9	1,640.3	52.19 ± 1.18	52.93	4.11 ± 0.03	4.08	51.02 ± 0.48
13	100	25	5	1,597 ± 5	1,602.1	54.39 ± 0.38	52.92	4.15 ± 0.04	4.06	51.32 ± 0.32
14	100	25	50	1,661 ± 9	1,644.4	57.38 ± 0.39	52.92	4.25 ± 0.02	4.11	52.11 ± 0.11
15	50	45	27.5	1,650 ± 5	1,640.6	70.84 ± 0.84	71.89	5.41 ± 0.04	5.42	66.84 ± 0.84
16	100	65	5	1,665 ± 6	1645.1	62.37 ± 0.37	62.82	4.43 ± 0.02	4.43	55.12 ± 0.62
17	0	65	5	1,587 ± 5	1,610.7	49.67 ± 0.68	50.96	3.74 ± 0.02	3.63	54.78 ± 0.73
18	50	45	5	1,589 ± 4	1,584.8	70.48 ± 0.48	71.90	5.05 ± 0.05	5.31	64.21 ± 0.23
19	50	65	27.5	1,700 ± 10	1,701.3	76.84 ± 1.34	75.19	5.66 ± 0.03	5.47	72.38 ± 0.68
20	0	65	27.5	1,711 ± 7	1,684.1	48 ± 1	50.95	3.60 ± 0.04	3.83	52.4 ± 0.41

Equation 5, which is driven from RSM for estimating the OSI of the soybean oil via adding *M*. *piperita* extract, is presented as below:

(5) $$\text{OSI = 3}.002‐0\text{.00378}\;X_{1}+0.0326\;X_{2}+0.05191\;X_{3}‐0.000365\;X_{2}^{2}‐0.000494\;X_{3}^{2}+0.000197\;X_{1}*X_{2}+0.000086\;X_{2}*X_{3}$$

**FIGURE 4:**
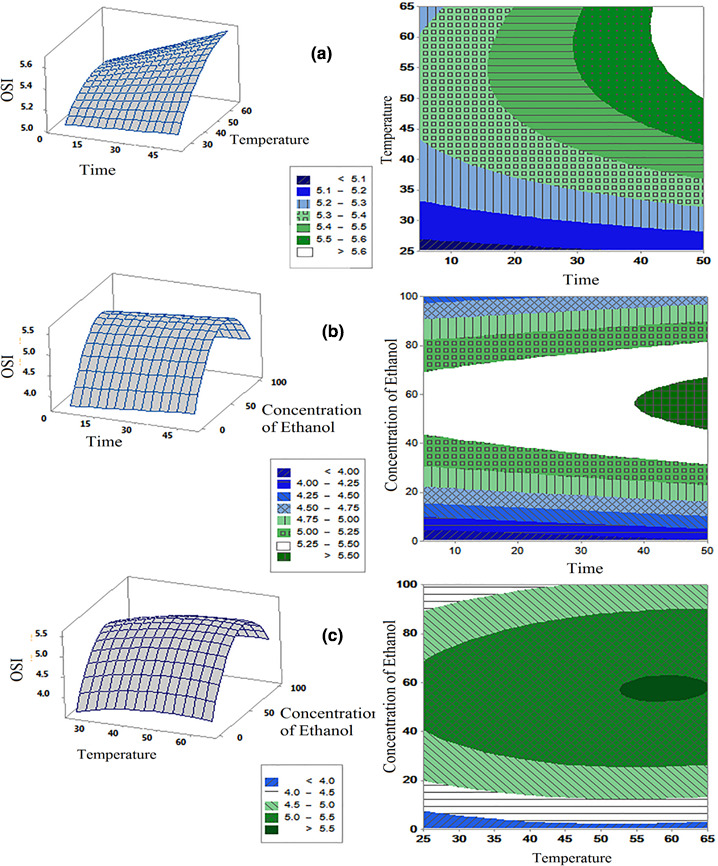
Response and contour plot for the effect of different parameters (time [min] and temperature [°C] (a), time [min] and concentration of ethanol [%] (b) and temperature [°C] and concentration of ethanol [%] (c)) on the amount of oxidative stability index (OSI, hr)

This model shows the same as phenol compound assay, all of the variables consist of time, temperature, and the ratio of ethanol to water, significantly affect the OSI test results. In contrast, the temperature and ethanol concentration have quadratic, linear, and interaction effects on the OSI. The time has linear and interaction effects with temperature. Due to the quadratic effect of the temperature and its linear and interaction with two other parameters can be considered the most crucial treatment for optimizing OSI of soybean oil with the *M*. *piperita* extract. Ethanol concentration and time were considered as the second and third important parameters, respectively. The interaction effect of binary variables on the optimization of OSI of *M*. *piperita* extract is presented in Figure 4. Probing the effect of time and temperature parameters on OSI of soybean oil (Figure 4A) indicated the optimum time and temperature for OSI assay was 50 min and 59.1°C, respectively. The interaction effect of time and ethanol concentration showed that the best time and ethanol concentration for OSI was 50 min and ethanol 59.1%, respectively (Figure 4B). Also, evaluating the interaction effect of temperature and ethanol concentration shows the optimum temperature, and ethanol concentration for OSI assay was 65°C and ethanol concentration of 57.6%, respectively (Figure 4C).

##### Optimization of OSI via three variables of time, temperature, and ethanol concentration

The optimum condition of three independent variables of time, temperature, and ethanol concentration on the OSI test was 50 min, 65°C, and ethanol concentration of 58.6% (Figure 2). This test, unlike the phenolic compounds extraction test, had optimum conditions at lower ethanol concentrations. This result was due to the lower extraction of phenolic compounds and consequently no creating the peroxidative conditions (similar to the free radical scavenging test). In limited researches, Rancimat assay has been considered as a dependent parameter in RSM methodology. It was found that the best OSI in soybean oil was obtained at 34.1 min, 52.9°C, and 53.5% ethanol concentration in water–ethanol solvent (Estakhr et al., 2019) that was different from the results of the present study. This difference may also be related to the nature of the two types of plants and compounds extracted. Taghvaei et al. (2012) studied the effect of the microwave, moisture content, and the ratio of solvent to dry substance on the chemical properties of extracted oil from the cottonseed. Their results indicated 3.57‐min exposure to microwave, moisture content of 14%, and the ratio of solvent to dry substance of 4 to 1 lead to best OSI (11.5 hr at 110°C).

#### 3.2.4 Determination of the final optimal conditions by simultaneously considering three tests of phenol compound extraction, DPPH radical scavenging assay, and OSI (Rancimat test)

Considering three independent variables of temperature, time, and ethanol concentration and the results of three phenol compounds extraction, DPPH radical scavenging assay, and Rancimat test, indicated the optimum condition was 50 min, 65°C, and the ethanol concentration of 59.6% (Figure 2). The final optimum condition was close to the optimum condition of the DPPH radical scavenging assay and Rancimat test. As can be seen, two parameters of temperature and time at the highest amount and the parameter of ethanol concentration around its mean value have the optimum effect.

#### 3.2.5 Validation of examinations

**FIGURE 5:**
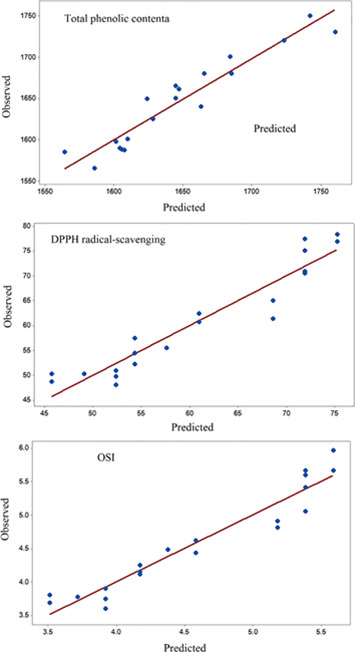
Comparison of the observed and predicted values of total phenolic content, DPPH radical scavenging, and oxidative stability index (OSI)

In response to surface methodology, in the validation step, the results obtained in the experimental phase were compared statistically with the derived amounts from the model. Comparing the results (Y0) and derived amounts from the model (Y) in Figure 5 and the results of Table 2 showed a very good correlation between them. Also, based on Figure 5, the regression coefficient of the curve of extracted phenol compounds, DPPH radical scavenging assay, and OSI test was 0.91, 0.91, and 0.92, respectively.

## 4 CONCLUSION

In this study, the optimization of polyphenol compound extraction and antioxidant activity (DPPH radical scavenging assay, FRAP test, and rancimat test [OSI]) of *M*. *piperita* plant via response surface methodology (RSM) was investigated. Therefore, three independent variables of time, temperature, and concentration of ethanol on the mention tests were studied. The results showed that all independent factors used in this study have no significant effect (*p*‐value >.05) on FRAP test of *M*. *piperita* extract, while the reverse results were observed for the other tests. Between the three RSM models that had been done to predict the results of the mentioned tests, the OSI model got the most accurate results, and the model for DPPH radical scavenging assay got the least accurate results.

Our results showed that the best conditions (final optimal conditions) were obtained at 50 min, 65°C, and the ethanol concentration of 59.6% by considering the three experiments used in this study. It was observed that the optimum concentration of ethanol was different in various tests. The optimum ethanol concentration for phenol compound extraction was 100%, while the other tests vary between 58.6% and 59.6%. Statistical analysis of the results shows ethanol concentration was the most influential parameter. Finally, the results' validation shows a good correlation between the experimental results and extracted data from the models.

## CONFLICT OF INTEREST

The authors declare no conflict of interest.

## AUTHOR CONTRIBUTIONS

**Fatemeh Royshanpour:** Conceptualization (equal); Data curation (equal); Formal analysis (equal); Funding acquisition (equal); Investigation (equal); Methodology (equal); Project administration (equal); Resources (equal); Software (equal); Supervision (equal); Validation (equal); Visualization (equal); Writing‐original draft (equal); Writing‐review & editing (equal). **Javad Tavakoli:** Conceptualization (equal); Data curation (equal); Formal analysis (equal); Funding acquisition (equal); Investigation (equal); Methodology (equal); Project administration (equal); Resources (equal); Software (equal); Supervision (equal); Validation (equal); Visualization (equal); Writing‐original draft (equal); Writing‐review & editing (equal). **Faranak beigmohammadi :** Conceptualization (equal); Data curation (equal); Formal analysis (equal); Funding acquisition (equal); Investigation (equal); Methodology (equal); Project administration (equal); Resources (equal); Software (equal); Supervision (equal); Validation (equal); Visualization (equal); Writing‐original draft (equal); Writing‐review & editing (equal). **Shima Alaee:** Conceptualization (equal); Data curation (equal); Formal analysis (equal); Funding acquisition (equal); Investigation (equal); Methodology (equal); Project administration (equal); Resources (equal); Software (equal); Supervision (equal); Validation (equal); Visualization (equal); Writing‐original draft (equal); Writing‐review & editing (equal). **Amin Mousavi Khaneghah:** Conceptualization (equal); Data curation (equal); Formal analysis (equal); Funding acquisition (equal); Investigation (equal); Methodology (equal); Project administration (equal); Resources (equal); Software (equal); Supervision (equal); Validation (equal); Visualization (equal); Writing‐original draft (equal); Writing‐review & editing (equal).
